# Supplementary material for: Meta-QTL Analysis and Genes Responsible for Plant and Ear Height in Maize (Zea mays L.)
Source: Plants (Basel). 2025 Jun 24;14(13):1943. doi: 10.3390/plants14131943 (PMC12251859; doi:10.3390/plants14131943)
Supplement: Supplementary file 1 [file plants-14-01943-s001.zip › plants-3692346-supplementary.pdf]

Table S1. The relevant candidate genes identified within MQTL.

MQTL	Gene_ID	Chr.	Physical Interval (Mb)	Description
MQTL1–1	Zm00001d027313	1	2.78–2.78	Probable xyloglucan endotransglucosylase/hydrolase protein 32
	Zm00001d027317	1	2.82–2.83	Rolled leaf2
	Zm00001d027326	1	2.98–2.30	Actin–related protein 4
	Zm00001d027368	1	3.81–3.81	Xyloglucan endotransglucosylase/hydrolase protein 5
	Zm00001d027373	1	3.96–3.96	Cell division control protein2 homolog
	Zm00001d027376	1	3.97–3.99	Cyclin–T1–5
	Zm00001d027431	1	5.12–5.13	Knotted related homeobox1
MQT1–2	Zm00001d027837	1	14.70–14.70	Cytochrome P450 704B1
	Zm00001d027854	1	15.30–15.31	Sucrose transporter1
MQTL1–3	Zm00001d028392	1	33.15–33.15	Actin depolymerizing factor5
	Zm00001d028395	1	33.17–33.17	Fructose–bisphosphate aldolase 3 chloroplastic
	Zm00001d028401	1	33.65–33.65	Auxin transporter–like protein 3
	Zm00001d028419	1	34.33–34.33	Cytochrome P450 family 76 subfamily C polypeptide 7
	Zm00001d028434	1	34.93–34.93	Cryptochrome–1
	Zm00001d028472	1	35.97–35.97	Far–red impaired responsive (FAR1) family protein
	Zm00001d028508	1	37.47–37.48	Sucrose synthase 3
	Zm00001d028521	1	38.04–38.04	Cyclin–dependent kinase B1–1
	Zm00001d028524	1	38.08–38.08	Ethylene–responsive transcription factor ERF014
MQTL1–4	Zm00001d029656	1	81.67–81.68	Actin–depolymerizing factor
	Zm00001d029674	1	82.23–82.24	photosynthetic electron transport2
	Zm00001d029679	1	82.47–82.47	Ethylene–responsive transcription factor ERF025
	Zm00001d029688	1	82.83–82.83	Cytochrome P450 family 704 subfamily A polypeptide 2
MQTL1–5	Zm00001d030894	1	166.19–166.20	Cyclin–D5–1
	Zm00001d030975	1	170.33–170.34	Indole–3–pyruvate monooxygenase YUCCA2
	Zm00001d030976	1	170.45–170.45	Cytochrome P450 CYP76M15
	Zm00001d030993	1	172.41–172.42	Auxin–responsive protein IAA4
	Zm00001d030994	1	172.45–172.45	KNOX2 domain
	Zm00001d031064	1	175.37–175.38	Auxin response factor 6
MQTL1–6	Zm00001d032651	1	233.13–233.14	Cytochrome P450 86B1
	Zm00001d032664	1	233.55–233.55	Cytokinin oxidase6
	Zm00001d032683	1	234.27–234.27	Auxin response factor 16
	Zm00001d032732	1	236.51–236.51	Cyclin–dependent kinase inhibitor1
	Zm00001d032776	1	237.89–237.89	Cellulose synthase10
	Zm00001d032784	1	238.04–238.04	Two–component response regulator ARR12
	Zm00001d032791	1	238.23–238.24	Xyloglucan galactosyltransferase MUR3
	Zm00001d032794	1	238.32–238.33	Xyloglucan galactosyltransferase MUR3
	Zm00001d032795	1	238.33–238.33	Probable xyloglucan galactosyltransferase GT15
	Zm00001d032797	1	238.40–238.40	Probable xyloglucan galactosyltransferase GT15

	Zm00001d032823	1	239.30–239.30	Glycine–rich cell wall structural protein 2
	Zm00001d032859	1	240.51–240.52	Cell division control protein 48 homolog D
	Zm00001d032876	1	241.26–241.26	Expansin–A11
	Zm00001d032881	1	241.34–241.34	Expansin–A11
	Zm00001d032882	1	241.34–241.34	Expansin–A11
	Zm00001d032883	1	241.40–241.40	Expansin–A11
	Zm00001d032884	1	241.40–241.41	Expansin–A11
	Zm00001d032885	1	241.44–241.44	Expansin–A11
	Zm00001d032886	1	241.48–241.48	Expansin–A11
	Zm00001d032906	1	242.70–242.70	Sugar transport protein 3
	Zm00001d032909	1	242.79–242.80	Cellulose synthase–like protein D3
MQTL2–3	Zm00001d005729	2	185.05–185.06	Cytokinin hydroxylase
	Zm00001d005737	2	185.42–185.42	Wavy auricles in blades1
	Zm00001d005775	2	187.82–187.83	Cellulose synthase A catalytic subunit 7 [UDP– forming]
MQTL3–1	Zm00001d039589	3	8.52–8.53	Gigantea2
	Zm00001d039624	3	9.37–9.37	Auxin–responsive protein IAA15
	Zm00001d039634	3	9.75–9.75	Dwarf plant1
	Zm00001d039635	3	9.81–9.81	BES1/BZR1 homolog protein 4
MQTL3–2	Zm00001d040379	3	40.80–40.80	Expansin–A14
	Zm00001d040477	3	45.76–45.76	Cyclin–like F–box
	Zm00001d040485	3	46.29–46.29	Light–mediated development protein DET1
	Zm00001d040509	3	47.52–47.52	Beta tubulin5
	Zm00001d040541	3	48.78–48.78	Auxin–responsive protein IAA20
MQTL3–3	Zm00001d042199	3	156.00–156.00	Photosystem II reaction center PSB28 protein chloroplastic
	Zm00001d042257	3	157.57–157.57	Photosynthetic NDH subunit of subcomplex B 5 chloroplastic
	Zm00001d042267	3	158.10–158.10	Auxin response factor 10
	Zm00001d042276	3	158.61–158.61	Probable beta–14–xylosyltransferase IRX10L
	Zm00001d042281	3	158.68–158.68	Secondary cell wall–related glycosyltransferase family 47
	Zm00001d042292	3	159.33–159.33	SAUR–like auxin–responsive protein family
	Zm00001d042315	3	160.59–160.59	MADS–box transcription factor 56
MQTL3–4	Zm00001d042946	3	184.67–184.67	Pectin lyase–like superfamily protein
MQTL3–5	Zm00001d043515	3	202.40–202.40	IAA6–auxin–responsive Aux/IAA family member
MQTL3–6	Zm00001d044396	3	227.10–227.10	Chlorophyll a–b binding protein 48%2C chloroplastic
	Zm00001d044399	3	227.15–227.15	Photosystem II light harvesting complex gene B1B2
	Zm00001d044401	3	227.19–227.19	Photosystem II light harvesting complex gene B1B2
	Zm00001d044402	3	227.28–227.28	Chlorophyll a–b binding protein 2
	Zm00001d044406	3	227.34–227.34	Two–component response regulator–like APRR1
	Zm00001d044494	3	229.64–229.65	Pectin acetylesterase 9
MQTL4–1	Zm00001d051340	4	155.23–155.23	Ethylene–responsive transcription factor ERF105
	Zm00001d051368	4	156.13–156.13	Expansin–B4
	Zm00001d051384	4	156.55–156.55	Cortical cell–delineating protein
	Zm00001d051388	4	156.85–156.85	Actin–depolymerizing factor 7
	Zm00001d051456	4	159.32–159.32	Growth–regulating factor

	Zm00001d051554	4	162.65–162.66	Abscisic acid 8'–hydroxylase2
	Zm00001d051556	4	162.74–162.74	Nine–cis–epoxycarotenoid dioxygenase6
	Zm00001d051584	4	163.36–163.37	Ethylene response protein
	Zm00001d051612	4	164.55–164.55	Gibberellin receptor GID1
MQTL4–2	Zm00001d053288	4	224.31–224.32	Fasciclin–like arabinogalactan protein 6
	Zm00001d053311	4	225.55–225.55	SAUR–like auxin–responsive protein family
	Zm00001d053320	4	225.98–225.98	Probable xyloglucan endotransglucosylase/hydrolase protein 26
	Zm00001d053363	4	227.74–227.74	Cyclin–dependent kinase B1–1
	Zm00001d053366	4	227.75–227.76	Cyclin–dependent kinase B1–1
	Zm00001d053395	4	229.17–229.17	Pectin lyase–like superfamily protein
	Zm00001d053396	4	229.22–229.22	Abscisic acid receptor PYL9
MQTL5–1	Zm00001d017397	5	194.71–194.71	SAUR11–auxin–responsive SAUR family member
	Zm00001d017415	5	195.35–195.35	Pectin lyase–like superfamily protein
	Zm00001d017416	5	195.35–195.36	Pectin lyase–like superfamily protein
	Zm00001d017429	5	195.83–195.84	Iron–phytosiderophore transporter yellow stripe 1
	Zm00001d017441	5	195.919–195.91	Cyclin–U4–2
	Zm00001d017462	5	196.59–196.59	Ethylene–responsive transcription factor 1A
	Zm00001d017466	5	196.74–196.74	Ethylene–responsive transcription factor ERF105
	Zm00001d017477	5	197.15–197.15	Ethylene–responsive transcription factor ERF035
	Zm00001d017478	5	197.22–197.22	Ethylene–responsive transcription factor ERF035
	Zm00001d017480	5	197.25–197.25	Ethylene–responsive transcription factor ERF035
	Zm00001d017493	5	197.52–197.52	Expansin–B4
	Zm00001d017494	5	197.53–197.53	Expansin–B4
	Zm00001d017495	5	197.54–197.54	Expansin–B4
	Zm00001d017508	5	197.91–197.91	Cortical cell–delineating protein
	Zm00001d017510	5	198.00–198.00	Cortical cell–delineating protein
	Zm00001d017516	5	198.49–198.49	Actin–depolymerizing factor
	Zm00001d017553	5	199.39–199.40	BZIP transcription factor
	Zm00001d017591	5	200.87–200.87	Ethylene–responsive transcription factor ERF025
	Zm00001d017612	5	201.606–201.60	Brassinosteroid–responsive RING–H2
	Zm00001d017725	5	205.29–205.30	Cell division cycle 20.1 cofactor of APC complex
	Zm00001d017731	5	205.53–205.53	Expansin–B4
	Zm00001d017732	5	205.53–205.53	Expansin–B4
	Zm00001d017733	5	205.53–205.54	Expansin–B4
	Zm00001d017734	5	205.54–205.54	Expansin–B4
	Zm00001d017735	5	205.56–205.56	Expansin–B4
	Zm00001d017736	5	205.58–205.58	Expansin–B4
	Zm00001d017737	5	205.58–205.58	Expansin–B4
	Zm00001d017740	5	205.62–205.62	Expansin–B4
	Zm00001d017742	5	205.73–205.74	Growth–regulating factor 6
	Zm00001d017743	5	205.75–205.75	Cell division control protein 48 homolog D
	Zm00001d017762	5	206.14–206.14	Abscisic acid 8'–hydroxylase1
	Zm00001d017766	5	206.20–206.20	Nine–cis–epoxycarotenoid dioxygenase8

	Zm00001d017782	5	206.60–206.60	SANT/MYB protein
MQTL6–1	Zm00001d037969	6	143.84–143.84	Inhibitor I family protein; Potato inhibitor I family
	Zm00001d037984	6	144.38–144.39	Chlorophyll synthase chloroplastic
MQTL7–1	Zm00001d019681	7	50.68–50.69	Cyclin–L1–1
	Zm00001d019684	7	50.70–50.71	Cyclin–L1–1
	Zm00001d019696	7	51.34–51.34	Cyclin10
	Zm00001d019734	7	54.54–54.54	Ethylene–responsive transcription factor 1B
	Zm00001d019744	7	55.35–55.35	Ethylene–responsive transcription factor 14
	Zm00001d019756	7	56.70–56.70	Beta–amylase
	Zm00001d019758	7	56.87–56.87	Chlorophyllase1
	Zm00001d019881	7	72.86–72.88	Auxin transport protein BIG
MQTL7–2	Zm00001d021435	7	152.28–152.28	Chlorophyll a–b binding protein 1%2C chloroplastic
	Zm00001d021448	7	152.39–152.39	Root hair specific 17
	Zm00001d021450	7	152.44–152.45	Cytokinin riboside 5'–monophosphate phosphoribohydrolase LOG5
	Zm00001d021454	7	152.61–152.61	SAUR55–auxin–responsive SAUR family member
	Zm00001d021455	7	152.64–152.64	SAUR52–auxin–responsive SAUR family member
	Zm00001d021456	7	152.64–152.65	SAUR55–auxin–responsive SAUR family member
	Zm00001d021457	7	152.65–152.65	Auxin–responsive protein SAUR61
	Zm00001d021459	7	152.72–152.72	Auxin–responsive protein SAUR61
	Zm00001d021497	7	154.12–154.12	Actin–depolymerizing factor 1
MQTL8–1	Zm00001d010159	8	102.41–102.42	Actin–1
	Zm00001d010175	8	103.05–103.05	Ethylene–responsive transcription factor ABR1
	Zm00001d010193	8	103.47–103.47	Gibberellin–regulated protein 2
	Zm00001d010247	8	105.89–105.89	Phenylalanine ammonia–lyase 1
	Zm00001d010275	8	107.05–107.06	Beta tubulin2
	Zm00001d010308	8	108.95–108.96	Gibberellin receptor GID1A
	Zm00001d010360	8	111.04–111.04	Auxin–responsive protein IAA26
MQTL8–2	Zm00001d011086	8	138.47–138.47	Actin–7
	Zm00001d011087	8	138.48–138.48	Actin–7
	Zm00001d011218	8	143.28–143.28	Light harvesting complex photosystem II subunit 6
	Zm00001d011225	8	143.69–143.69	ZmGR2c–like
	Zm00001d011246	8	144.41–144.41	Cytokinin riboside 5'–monophosphate phosphoribohydrolase LOG7
	Zm00001d011285	8	145.83–145.83	Photosystem II light harvesting complex gene B1B2
MQTL8–3	Zm00001d012175	8	169.55–169.55	Pectin acetylesterase 5
	Zm00001d012180	8	169.62–169.62	Ig1–as2 like1
	Zm00001d012212	8	170.12–170.12	Gibberellin 20–oxidase5
	Zm00001d012222	8	170.39–170.39	SAUR–like auxin–responsive protein family
MQTL9–1	Zm00001d046501	9	92.85–92.85	Ethylene–responsive transcription factor WIN1
	Zm00001d046568	9	96.27–96.28	Knotted related homeobox2
	Zm00001d046691	9	102.51–102.52	Cellulose synthase A catalytic subunit 5 [UDP– forming]
	Zm00001d046723	9	103.58–103.58	Expansin–A20
	Zm00001d046755	9	104.44–104.45	Putative two–component response regulator family protein
	Zm00001d046786	9	105.96–105.96	Photosystem I chlorophyll a/b–binding protein 3–1 chloroplastic

	Zm00001d046893	9	109.34–109.34	Auxin efflux carrier component 2
MQTL10–1	Zm00001d023398	10	4.73–4.73	Probable xyloglucan 6–xylosyltransferase 5
	Zm00001d023399	10	4.73–4.73	Probable xyloglucan 6–xylosyltransferase 5
	Zm00001d023417	10	5.09–5.09	Probable xyloglucan 6–xylosyltransferase 5
	Zm00001d023418	10	5.09–5.09	Probable xyloglucan 6–xylosyltransferase 5
MQTL10–2	Zm00001d023659	10	13.88–13.88	Auxin response factor 2
	Zm00001d023664	10	14.45–14.45	ABA–responsive protein
	Zm00001d023736	10	18.03–18.04	Agamous–like MADS–box protein AGL8
	Zm00001d023760	10	19.08–19.08	Cell division topological specificity factor homolog chloroplastic
	Zm00001d023838	10	23.89–23.90	Auxilin–related protein 1
MQTL10–3	Zm00001d024379	10	68.16–68.16	Xyloglucan endotransglucosylase/hydrolase protein 2
	Zm00001d024382	10	68.27–68.27	Xyloglucan endotransglucosylase/hydrolase protein 2
	Zm00001d024386	10	68.32–68.33	Xyloglucan endo–transglycosylase/hydrolase1
	Zm00001d024389	10	68.37–68.37	Putative MADS–box transcription factor family protein
	Zm00001d024392	10	68.48–68.48	Probable xyloglucan endotransglucosylase/hydrolase protein 21
	Zm00001d024393	10	68.58–68.59	Indole–3–glycerol phosphate synthase chloroplastic
	Zm00001d024468	10	72.23–72.23	AP2–like ethylene–responsive transcription factor AIL1
MQTL10–4	Zm00001d024913	10	94.66–94.66	Pectin methylesterase inhibitor 1
	Zm00001d025018	10	99.92–99.92	Expansin–A11
	Zm00001d025036	10	100.77–100.78	Indole–3–acetate beta–glucosyltransferase
	Zm00001d025050	10	101.68–101.68	Ethylene receptor homolog2

Chr.: Chromosome.
